# Intra-Articular Administration of Cramp into Mouse Knee Joint Exacerbates Experimental Osteoarthritis Progression

**DOI:** 10.3390/ijms22073429

**Published:** 2021-03-26

**Authors:** Moon-Chang Choi, Jiwon Jo, Myeongjin Lee, Jonggwan Park, Yoonkyung Park

**Affiliations:** 1 Department of Biomedical Science, Chosun University, Gwangju 61452, Korea; choist777@chosun.ac.kr (M.-C.C.); ehklwl55@chosun.ac.kr (J.J.); ksf9696@chosun.ac.kr (M.L.); 2 Department of Bioinformatics, Kongju National University, Kongju 38065, Korea; jgpark@kongju.ac.kr

**Keywords:** osteoarthritis, cramp, cathelicidin, cartilage degeneration, meniscus ossification

## Abstract

Osteoarthritis (OA) is the most common type of arthritis and is associated with wear and tear, aging, and inflammation. Previous studies revealed that several antimicrobial peptides are up-regulated in the knee synovium of patients with OA or rheumatoid arthritis. Here, we investigated the functional effects of cathelicidin-related antimicrobial peptide (Cramp) on OA pathogenesis. We found that Cramp is highly induced by IL-1β via the NF-κB signaling pathway in mouse primary chondrocytes. Elevated Cramp was also detected in the cartilage and synovium of mice suffering from OA cartilage destruction. The treatment of chondrocytes with Cramp stimulated the expression of catabolic factors, and the knockdown of Cramp by small interfering RNA reduced chondrocyte catabolism mediated by IL-1β. Moreover, intra-articular injection of Cramp into mouse knee joints at a low dose accelerated traumatic OA progression. At high doses, Cramp affected meniscal ossification and tears, leading to cartilage degeneration. These findings demonstrate that Cramp is associated with OA pathophysiology.

## 1. Introduction

Osteoarthritis (OA) is a joint disorder that affects most of the joint components, such as by causing articular cartilage degeneration, synovial inflammation, subchondral bone sclerosis, osteophyte formation and/or meniscal tear [1]. This degenerative joint disease is caused by overuse and overload of the joints together with joint inflammation, and leads to pain and disability mainly in the aging and obese populations [2]. Acute or degenerative meniscal lesions that decrease knee joint stability are also associated with disease development in patients with OA, including relatively young individuals [3,4,5]. OA treatment is currently limited to pain control without licensed disease-modifying OA drugs. Defining OA-associated factors may provide biomarkers and therapeutic targets.

Cartilage homeostasis is maintained by a balance between chondrocyte anabolism and catabolism. Chondrocytes, the only cell type present in cartilage, normally produce a large amount of anabolic extracellular matrix (ECM) proteins, such as Col2a1 and aggrecan, to tolerate shearing forces and absorb shock [6]. However, in OA disease, prolonged mechanical stress and synovial inflammation promote chondrocyte catabolism, leading to ECM degradation and breakdown in the cartilage [7,8,9]. The infrapatellar fat pad also contributes to OA pathogenesis by producing pro-inflammatory mediators [10]. Mechanistically, chondrocytes stimulated by pro-inflammatory cytokines or ECM degradation products from OA lesions express high levels of catabolic factors such as matrix metalloproteinases (MMPs) and aggrecanases (ADAMTSs) [11,12,13], which degrade the cartilage matrix. The induction of these catabolic genes occurs via several signaling pathways, including NF-κB transcription factor and associated IκBζ [14,15]. The genetic ablation of these genes in chondrocytes can protect against OA development [15,16]. Hence, identifying factors that regulate catabolic pathways can provide potential therapeutic targets for OA.

The meniscus plays integral roles in preserving joint health and preventing OA lesions. Due to its shock absorption capability, it is well-accepted that pathologic changes in menisci due to injury predispose individuals to OA development [17,18], and thus meniscus damage is considered to be a risk factor for OA. Although the mouse knee joint normally shows small areas of ossification in the anterior meniscal horn [19], either traumatic or spontaneous OA leads to severe ossification of the mouse meniscus [20]. In other animals, such as guinea pigs, dogs, and pigs, meniscus ossification is also closely associated with OA cartilage damage [21,22,23,24,25]. However, compared with the understanding of OA pathogenesis related to cartilage degeneration or synovial inflammation, the factors associated with OA meniscal damage are poorly understood.

Antimicrobial peptides (AMPs), also known as host defense peptides, are components of innate immunity and play critical roles in protecting against bacterial, viral, and fungal infections [26]. Two major AMPs in mammals are cathelicidins and defensins, which have broad-range antimicrobial activities [27,28]. The only cathelicidin protein identified to date in humans is LL-37. This short peptide is derived from the precursor protein hCAP18 via proteolytic cleavage [29]. Cathelicidin-related antimicrobial peptide (Cramp), the rodent homologue of LL-37, also shows potent antibiotic activity and is expressed in many cell types such as neutrophils [30,31], adipocytes [32], macrophages [33], and epithelial cells [34]. In addition to their roles in infectious diseases, cathelicidins have pleiotropic effects in non-infectious diseases such as acute thrombosis [30], atherosclerosis [31], carcinogenesis [35], kidney injury [36], cardiac dysfunctions [37,38], and liver injury [39] by exerting either disease-causative or protective roles.

Interestingly, several AMPs, including LL-37 and human beta-defensin 3 (HBD-3), have been implicated in the pathogenesis of arthritic diseases. In fact, it has long been recognized that LL-37 is elevated in the synovial membrane of patients with OA and rheumatoid arthritis (RA) [40]. More recent reports supported the up-regulation of cathelicidins in the RA synovium [41,42,43]. However, whether cathelicidins function in the development of OA remains unknown. Therefore, the objective of this study was to investigate whether Cramp is involved in OA pathogenesis.

## 2. Results

### 2.1. Cramp Is Induced by Pro-Inflammatory Cytokines via NF-κB Pathway in Chondrocytes

**Figure 1:**
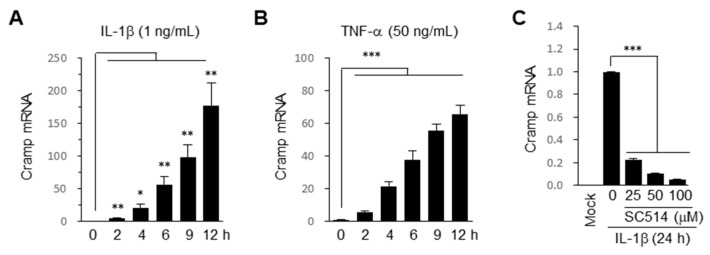
Cathelicidin-related antimicrobial peptide (Cramp) expression is up-regulated via NF-κB signaling pathway in pro-inflammatory cytokine-treated chondrocytes. (**A**) Mouse primary chondrocytes were treated with IL-1β for the indicated concentrations and times. The mRNA level of Cramp was determined by quantitative PCR (*n* = 4). Values are the means ± SEM. * *p* < 0.05, ** *p* < 0.01 versus untreated 0 h. (**B**) Cramp mRNA was examined in chondrocytes treated with TNF-α for the indicated times (*n* = 4). (**C**) Chondrocytes pre-treated with SC514 for 1 h were treated with IL-1β for 24 h. The mRNA level of Cramp was determined by qPCR (*n* = 5). Values are the means ± SEM. *** *p* < 0.001.

Soluble mediators, such as pro-inflammatory IL-1β and TNF-α, secreted from damaged joints simulate catabolic gene expression in cultured chondrocytes and thus transform them into ECM-catabolizing cells [11]. We first analyzed whether Cramp expression is altered by IL-1β in mouse primary chondrocytes. The results showed that Cramp mRNA was rapidly and strongly induced by IL-1β in a time-dependent manner (Figure 1A). TNF-α also significantly increased the mRNA level of Cramp, but to a lesser extent compared to IL-1β (Figure 1B).

NF-κB induces many genes associated with OA cartilage degeneration [14]. To examine whether IL-1β-induced expression of Cramp is mediated by NF-κB, we treated chondrocytes with SC514, an IKKβ inhibitor [44], and observed significant suppression of Cramp induction (Figure 1C). This result suggests that pro-inflammatory IL-1β induces Cramp expression in an NF-κB-dependent manner during chondrocyte catabolism.

### 2.2. Cramp Expression Is Elevated in Cartilage and Synovium of Experimental OA

**Figure 2:**
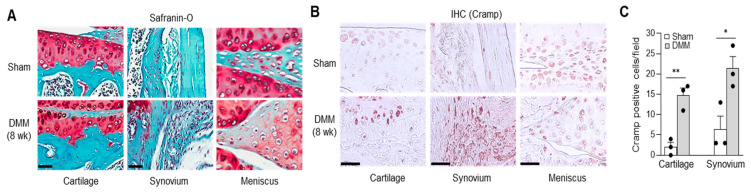
Cramp is up-regulated in cartilage and synovium during experimental osteoarthritis (OA). (**A**) Safranin-O staining of mouse cartilage, synovium and meniscus harvested at 8 weeks after destabilization of the medial meniscus (DMM) surgery. (**B**,**C**) Immunohistochemical staining (IHC) for Cramp in cartilage, synovium and meniscus. Bars = 50 μm. Values are the means ± SEM. *n* = 3 for each group. * *p* < 0.05, ** *p* < 0.01 versus sham.

We next investigated whether Cramp is indeed activated in joint components during OA development. Experimental OA was induced by subjecting mice to destabilization of the medial meniscus (DMM) surgery, which leads to ECM degradation and synovial inflammation (Figure 2A). Cramp levels in knee joints from sham-operated or DMM-operated mice were determined by immunohistochemistry (IHC) staining (Figure 2B,C). Compared to sham-operated joints, DMM-operated joints exhibited strong expression of Cramp in both the articular cartilage and synovium. Cramp levels in the meniscus were comparable between sham and DMM. This in vivo finding suggests that Cramp is activated in the cartilage and synovium during experimental OA progression.

### 2.3. Cramp Increases Mmp3 and Mmp13 Expression in Chondrocytes

**Figure 3:**
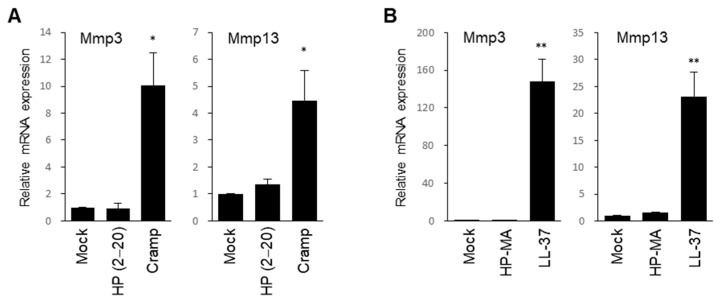
Treatment of Cramp or human LL-37 increases Mmp3 and Mmp13 expressions. (**A**) Mouse primary chondrocytes were treated with mock, control HP (2–20) peptide (40 μM), or Cramp peptide (40 μM) for 24 h. mRNA levels of Mmp3 and Mmp13 were determined by qPCR (*n* = 3). (**B**) Chondrocytes were treated with mock, control HP-MA peptide (10 μM), or LL-37 peptide (10 μM) for 24 h (*n* = 3). Values are the means ± SEM. * *p* < 0.05, ** *p* < 0.01 versus mock.

The up-regulation of Cramp in OA chondrocytes led us to investigate whether Cramp plays a role in chondrocyte catabolism. A recent report showed that mouse Cramp and human LL-37 effectively worked on platelet activation at >20 and >5 μM, respectively [30]. We therefore treated chondrocytes for 24 h with cathelicidins at 40 μM Cramp or 10 μM LL-37 and examined catabolic factor expression. Two types of synthetic AMPs, HP (2–20) and HP-MA, which exert potent antimicrobial activities [45,46], were used as control peptides. Compared to mock or control HP (2–20) peptide-treated groups, Cramp-treated chondrocytes showed higher expression of Mmp3 and Mmp13 (Figure 3A). LL-37 potently induced Mmp3 and Mmp13 (Figure 3B). Considering the crucial role of Mmp13 in OA matrix degradation [47], the increase in Mmp13 by Cramp suggested that Cramp might be associated with OA development.

### 2.4. siRNA-Mediated Knockdown of Cramp Reduces IL-1β-Induced Catabolic Gene Expression

**Figure 4:**
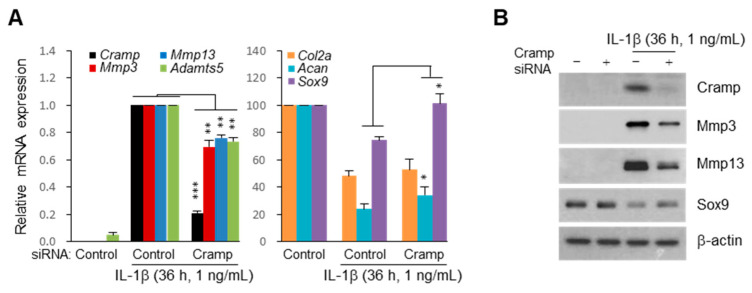
Small interfering RNA (siRNA) knockdown (KD) of Cramp attenuates catabolic factor expression induced by IL-1β. Chondrocytes transfected with control or Cramp siRNA were treated with IL-1β for 36 h. (**A**) mRNA levels of catabolic factors (left) and anabolic factors (right) were determined by qPCR (*n* = 4). Values are the means ± SEM. * *p* < 0.05, ** *p* < 0.01, *** *p* < 0.001. (**B**) Cellular Cramp, Sox9, and β-actin and extracellular Mmp3 and Mmp13 were determined by Western blotting. Data from one representative experiment of two independent experiments are shown.

To validate the function of Cramp in regulating matrix-degrading enzymes, we transiently inactivated Cramp by small interfering RNA (siRNA) knockdown (KD) in chondrocytes, and examined IL-1β-induced catabolic factor expression. As shown in Figure 4A (left), the expression of Mmp3, Mmp13, and Adamts5 induced by IL-1β was decreased by Cramp KD. We also found that the reductions in anabolic aggrecan and Sox9 by IL-1β were partially restored by Cramp KD (Figure 4A, right). Western blot analysis confirmed the reduction in Mmp3/13 and partial restoration of Sox9 in Cramp KD chondrocytes (Figure 4B). Collectively, these in vitro results indicate that Cramp is required for the proper activation of chondrocyte catabolism.

### 2.5. Cramp Exacerbates the Progression of Experimental OA

**Figure 5:**
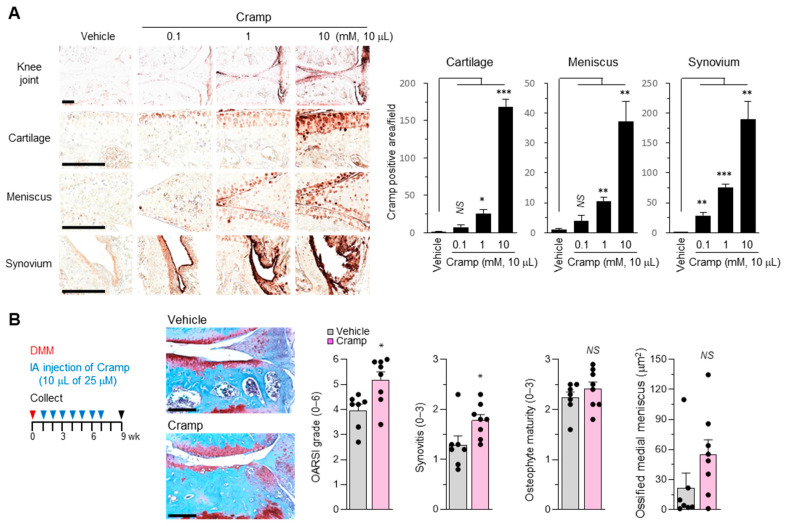
Cramp treatment into mouse knee joint by intra-articular (IA) injection promotes traumatic OA progression. (**A**) IHC staining of Cramp in mouse knee joints treated with indicated concentrations of Cramp for 24 h. Bars = 200 μm. Values are the means ± SEM. *n* = 3 for each group. * *p* < 0.05, ** *p* < 0.01, *** *p* < 0.001 versus vehicle; *NS*, not significant. (**B**) 12-week-old male mice were subjected to DMM surgery, and treated with either vehicle (*n* = 7) or Cramp (*n* = 8) for the indicated periods and concentrations. Safranin-O staining, Osteoarthritis Research Society International (OARSI) grade, synovial inflammation, osteophyte maturity, and meniscus ossification are shown. Values are the means ± SEM. * *p* < 0.05 versus vehicle-treated group. Bars = 200 μm.

The functional role of Cramp was further investigated in vivo following the intra-articular (IA) injection of Cramp. We first determined which joint components are targets affected by secreted Cramp. The degree of Cramp internalization into the joint components was monitored at 24 h after IA injection of increasing concentrations of Cramp. As shown in Figure 5A, when a relatively low dose of Cramp (10 μL of 0.1 mM) was IA-injected, Cramp was enriched in the synovial membrane. Higher concentrations of Cramp (10 μL of 1–10 mM) led to a dose-dependent internalization of Cramp with the cartilage and meniscus as well as the synovium. This finding suggests that Cramp might play a role in OA by associating with the cartilage, synovium, and meniscus.

To evaluate the pathological changes caused by Cramp in OA, we repeatedly treated mice with a low dose of Cramp under experimental OA conditions. As shown in Figure 5B, 12-week-old male mice were subjected to DMM surgery. At 1 week after DMM, the mice were first treated with either vehicle or Cramp (10 μL of 25 μM, approximately 1 μg) by IA injection, followed by weekly injections. Interestingly, safranin-O staining and Osteoarthritis Research Society International (OARSI) scoring revealed that Cramp-treated mice had more severe cartilage destruction and synovial inflammation than vehicle-treated mice. Although not statistically significant, meniscus ossification appeared to be slightly increased in Cramp-treated mice. Taken together with our in vitro data, these results suggest that Cramp promotes OA.

### 2.6. Introduction of Excessive Cramp Causes Meniscus Ossification and Tears

**Figure 6:**
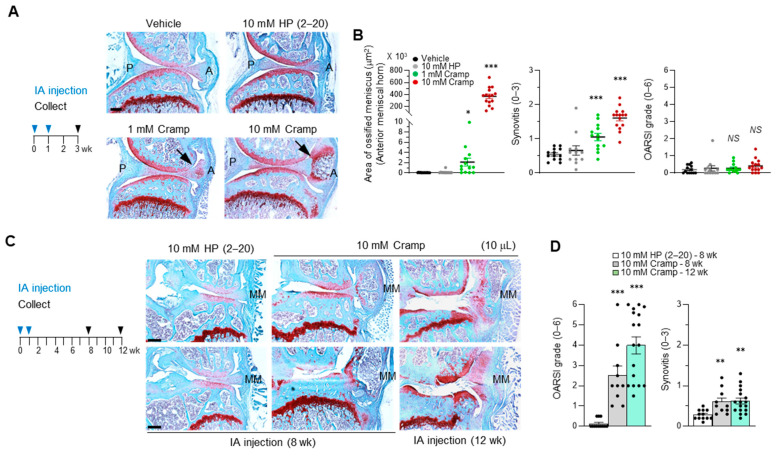
IA injection of Cramp at high concentrations results in ossification and tear of the meniscus and cartilage destruction. (**A**,**B**) 12-week-old male mice were subjected to IA injection with 10 μL of vehicle (*n* = 13), 10 mM control HP (2–20) peptide (*n* = 12), 1 mM Cramp (*n* = 13) or 10 mM Cramp (*n* = 15) for 3 weeks. (**A**) Safranin-O staining of cartilage sections. Arrows indicate the region of meniscal ossification. P, posterior meniscal horn; A, anterior meniscal horn. Bars = 200 μm. (**B**) Measurement of ossified region in anterior meniscal horn or scoring of synovitis and OARSI grade. Values are the means ± SEM. * *p* < 0.05, *** *p* < 0.001 versus vehicle-treated group. *NS*, not significant. (**C**,**D**) 12-week-old male mice were treated with 10 μL of 10 mM control HP (2–20) peptide or Cramp. Mice were harvested at 8 or 12 weeks after first IA injection. Representative images of safranin-O staining (**C**) and scoring of OARSI grade and synovitis (**D**) are shown. MM, medial meniscus. Bars = 200 μm. Values are the means ± SEM. *n* = 12 for 10 mM HP (2–20) 8wk, *n* = 10 for 10 mM Cramp 8wk and *n* = 17 for 10 mM Cramp 12wk. ** *p* < 0.01, *** *p* < 0.001 versus control HP (2–20) peptide-treated group.

We next investigated whether high concentrations of Cramp could lead to changes in the joint architecture. As shown in Figure 6A, mice were treated with 10 μL of 10 mM control HP (2–20) peptide, 1 mM Cramp, or 10 mM Cramp once weekly for two weeks. Histological evaluation was conducted with joint sections of mice collected three weeks after the first IA injection. Whereas the control 10 mM HP (2–20) peptide showed no visible effects on joint homeostasis, 1 mM Cramp caused mild to moderate ossification in the anterior location of the menisci and medial meniscus (Figure 6A). Measurement of the ossified region indicated a significant increase in the ossification of the anterior meniscal horn by 1 mM Cramp treatment (Figure 6B). Interestingly, excessive Cramp at 10 mM resulted in a large amount of ossification of the meniscus (Figure 6A,B). While cartilage destruction was not obvious at three weeks after Cramp treatment, Cramp dose-dependently increased synovial inflammation. These results indicate that Cramp causes pathological changes in the meniscus and synovium at the early period. 

Meniscus modifications, including ossification, have been reported in both spontaneous and traumatic OA [20,21,48]. We hypothesized that meniscal ossification induced by Cramp could change the joint biomechanics and cartilage destruction. Therefore, we examined the long-term effects of 10 mM Cramp on joint destruction. At 8 weeks, a large amount of ossification or breakdown of the medial meniscus and subsequent cartilage destruction were observed with elevated synovitis (Figure 6C,D). At 12 weeks, most joints showed meniscal tears with cartilage destruction. Thus, meniscal ossification and tears caused by Cramp influenced joint stability and thereby caused cartilage destruction. Collectively, these data indicate that Cramp is involved in various skeletal pathophysiologies, including chondrocyte catabolism in articular cartilage, inflammation in the synovial membrane and bone development in the meniscus.

## 3. Discussion

Increasing evidence has demonstrated that cathelicidins play a critical role not only in infectious diseases but also in controlling many types of tissue diseases. Although previous studies identified the up-regulation of LL-37 in OA synovial membranes [40], its biological effect was unknown. In this study, we examined the functional role of cathelicidin in murine OA pathogenesis and found that Cramp has disease-promoting roles in OA development by regulating chondrocyte catabolism, synovial inflammation and meniscus damage.

We initially observed a high level of Cramp in OA cartilage, suggesting that this AMP might participate in OA pathogenesis. The hypothesis was also based on studies of another AMP, HBD-3. Up-regulation of HBD-3 along with Cramp was found in the synovial membrane of patients with OA [40]. A subsequent study showed that HBD-3 is not only up-regulated in the cartilage of OA patients, but also stimulates the expression of catabolic MMPs in chondrocytes [49]. As these HBD-3 results were very similar to our findings in this study, we speculate that AMPs function as general factors for OA development, possibly by acting through cooperative mechanisms.

We found a significant increase in Cramp expression in traumatic OA cartilage and chondrocyte culture. The chondrocyte catabolism stimulated by Cramp treatment suggests that Cramp is associated with ECM degradation in the articular cartilage. Moreover, Cramp was found to be required for IL-1β-induced expression of matrix-degrading enzymes, including Mmp13 and Adamts5 (Figure 4A). As the genetic ablation of either Mmp13 or AdamtS5 prevents OA cartilage destruction [47,50], our findings support the causative role of Cramp in cartilage catabolism. However, studies are needed to determine whether Cramp-deficient mice also exhibit alleviation of OA development and how Cramp activates catabolic factor expression. Thus, the effects of Cramp should be confirmed using knockout mice and the associated signaling pathways should be defined to target OA development.

In histological analysis, the internalization of exogenous Cramp was monitored in mouse knee joints by IHC staining, and was detected in the synovium, cartilage, and meniscus even at 24 h after IA injection (Figure 5A). Due to the antibody sensitivity or Cramp half-life during the treatment period, it was difficult to determine how much Cramp was delivered into each joint component. Nevertheless, Cramp appeared to associate with cells in the synovial membrane, cartilage, and meniscus. Several receptors, including FPR2, P2 × 7, and CXCR2, have been proposed to interact with cathelicidins [28,29]. In addition, ECM- or caveolae-mediated mechanisms have been reported in the regulation of cathelicidin transportation [31,51,52]. Although the precise mechanisms need to be investigated, Cramp may be involved in catabolic signaling in the joint tissues through interactions with the above signaling mediators.

Meniscus degeneration and tears are key features of OA disease [53]. As studies of such soft tissue changes are considered to be important for understanding OA disease, the identification of factors that regulate meniscus homeostasis or degeneration can provide clues for developing strategies for treating OA disease. In this study, we evaluated the meniscus-disrupting factor Cramp. Our results demonstrated that ectopic introduction of approximately 40 μg of Cramp (10 μL of 1 mM Cramp) into mouse knee joints visibly increased meniscus ossification (Figure 6B). Furthermore, dose-dependent development of meniscus ossification and cartilage destruction were observed when very high and potentially artificial concentrations of Cramp (10 μL of 10 mM Cramp) were IA-injected. As the most notable change in the early period of Cramp-treated joints was an increase in meniscus ossification, the cartilage destruction observed might be due to abnormal bone formation in the meniscus followed by meniscus breakdown. Interestingly, recent studies identified several transcription factors, such as Foxo1, Foxo3, and Mohawk, which are critical for the maintenance of meniscus homeostasis and for reducing OA severity [54,55]. In fact, the genetic inhibition of Foxo1/3 resulted in increased meniscal ossification. Whether Cramp participates in functional cross-talk with the transcription factors in meniscus biology needs to be determined in further studies. It would also be interesting to investigate whether endogenous and exogenous Cramp promote osteogenesis in vitro.

## 4. Materials and Methods

### 4.1. Chondrocyte Culture, Peptide Treatment, and siRNA Transfection

Mouse primary chondrocytes were isolated from the femoral condyles and tibial plateaus of 5-day-old mice by digestion with 0.2% collagenase (Sigma, St. Louis, MO, USA; C6885) as described previously [15]. Chondrocytes (5–6 × 10^5^) were seeded into 6-well plates in Dulbecco’s Modified Eagle’s Medium containing 10% fetal bovine serum and antibiotics. The next day, the cells were serum-starved overnight, and then treated with each peptide for 24 h at the concentrations indicated in each figure legend. All peptides were synthesized by Anygen Inc. (Gwangju, Korea). The amino acid sequences of the four peptides were as follows: Cramp, GLLRKGGEKIGEKLKKIGQKIKNFFQKLVPQPEQ; LL-37, LLGDFFRKSKEKIGKEFKRIVQRIKDFLRNLVPRTES; HP (2–20), AKKVFKRLEKLFSKIQNDK; HP-MA, AKKVFKRLGIGKFLHSAKKF. IL-1β (GenScript, Piscataway, NJ, USA; Z02922), TNF-α (EMD Millipore, Temecula, CA, USA; GF027), and SC514 (Tocris Bioscience; 3318) were also used for cell experiments at the concentrations and periods indicated in the figure legends.

For KD analysis of Cramp, the cells were treated with hyaluronidase for 2 h in serum-free medium, and then transfected with either 10 nM of control or Cramp siRNA (Ambion, Austin, TX, USA) using Lipofectamine RNAiMAX (Invitrogen, Carlsbad, CA, USA; 13778150). Chondrocytes were harvested at 36 h after treatment with IL-1β.

### 4.2. RNA Analysis

Total RNA was extracted from the cells using TRI reagent (Molecular Research Center, Cincinnati, OH, USA). RNA was reverse-transcribed using oligo dT primer and reverse transcriptase (Promega, Madison, WI, USA; A3800) according to the manufacturer’s instructions. Quantitative PCR was performed using qPCR 2X PreMIX SYBR (Enzynomics, Daejeon, Korea; RT500) on a 7500 Real Time PCR System (Applied Biosystems, Foster City, CA, USA). Data were calculated using the 2^−ΔΔCt^ method. The values were normalized to β-actin levels. Primers were synthesized according to our previous report [15]. The Cramp primer sequences used were as follows: 5′-CTACCTGAGCAATGTGCCTTC-3′ and 5-CAGGCCTACTACTCTGGCTGA-3′.

### 4.3. Western Blot Analysis

Whole cell lysates were extracted in lysis buffer (50 mM Tris HCl, pH 7.4, 150 mM NaCl, 1 mM EDTA, 1% NP-40, 0.5% sodium deoxycholate) containing cocktails of protease and phosphatase inhibitors (Thermo Fisher Scientific, Waltham, MA, USA; 78444). Protein concentrations were determined by BCA assay (Sigma, St. Louis, MO, USA). Culture supernatants were also collected to determine the levels of extracellular Mmp3 and Mmp13. The lysates or supernatants were resolved by SDS-PAGE, transferred onto nitrocellulose membranes, and incubated with antibodies against Cramp (1:1000; Abcam, Cambridge, UK; ab93357), Mmp3 (1:10,000; Abcam; ab52915), Mmp13 (1:500; Aviva Systems Biology, San Diego, CA, USA; ARP56350_P050), Sox9 (1:1000; Abcam; ab185966), or β-actin (1:10,000; Abcam; ab8226).

### 4.4. IA Injection in Mice, and Experimental OA

Twelve-week-old male mice on the C57BL/6 background were used for animal experiments. For IA injection of the peptide into the mouse knee joint, the mice were anesthetized and injected with 10 μL of each peptide. The experimental design of IA injection with peptide concentration and injection period is shown in each figure. For the experimental OA study, DMM surgery was performed as described previously [56]. Seven days after DMM surgery, the vehicle or Cramp peptide (10 μL of 25 μM; approximately 1 μg) was first injected into mice by IA injection, and then injected weekly a total of seven times before the mice were sacrificed at 9 weeks for histological analysis. The mice were housed in the animal facility of Chosun University. All mouse procedures were approved by the Chosun University Institutional Animal Care and Use Committee (approval no. CIACUC2017-A0046).

### 4.5. Histology and Immunohistochemistry

To prepare paraffin blocks, the knee joints of mice were fixed in 4% paraformaldehyde for 24 h, decalcified in 0.5 M EDTA for 2–3 weeks, and embedded in paraffin. Blocks were sectioned at a thickness of 7 μM. Serial sections were obtained from the whole joint tissue, and 3–5 sections per mouse were stained with safranin-O as described previously [15]. Cartilage degeneration was scored by three observers blinded to the sample information using the OARSI grading system [57]. Synovitis and osteophyte maturity were scored as described previously [15]. The degree of meniscus ossification was determined by measuring the ossified area in the anterior meniscal horn using ImageJ software (NIH, Bethesda, MD, USA). For immunohistochemical staining of Cramp, antigen retrieval was conducted by incubating the sections with trypsin (Abcam; ab970) for 30 min. After blocking with 10% normal goat serum (Thermo Fisher Scientific; 50062Z), the sections were incubated with the Cramp antibody (Innovagen AB, Lund, Sweden; PA-CRPL-100) at 4 °C overnight. Using an IHC staining kit (GBI Labs, Bothell, WA, USA; D06), the sections were incubated with secondary antibody followed by incubation with chromogen AEC according to the manufacturer’s instructions. Cramp levels were measured using ImageJ software.

### 4.6. Statistical Analysis

All data collected in this study were analyzed statistically by Student’s two-tailed *t*-test using the Microsoft Office 365 Excel application. The number of independent experiments (cell experiments) or mice is indicated by *n*. The results were considered to be significant when *p* < 0.05.

## 5. Conclusions

In this study, we explored whether Cramp regulates OA pathogenesis. We found that Cramp, an up-regulated peptide in OA disease, promotes chondrocyte catabolic functions. We further showed that the administration of Cramp into mouse knee joints accelerates experimental OA progression and meniscal damage. Overall, our findings suggest that Cramp plays a role in OA disease.

## Data Availability

The data will be available from the corresponding author upon reasonable request.
